# Objectives, methods, and results in critical health systems and policy research: evaluating the healthcare market

**DOI:** 10.1186/s12913-020-05889-w

**Published:** 2020-12-09

**Authors:** Jean-Pierre Unger, Ingrid Morales, Pierre De Paepe

**Affiliations:** 1 Department of Public Health, Institute of Tropical Medicine, Nationalestraat 155, B-2000 Antwerp, Belgium; 2 Medical Director, Office de la Naissance et de l’Enfance, French Community of Belgium, Chaussée de Charleroi 95, B-1060 Brussels, Belgium

**Keywords:** Health policy, Health systems research, Research methodology

## Abstract

**Background:**

Since the 1980s, markets have turned increasingly to intangible goods – healthcare, education, the arts, and justice. Over 40 years, the authors investigated healthcare commoditisation to produce policy knowledge relevant to patients, physicians, health professionals, and taxpayers. This paper revisits their objectives, methods, and results to enlighten healthcare policy design and research.

**Main text:**

This paper meta-analyses the authors’ research that evaluated the markets impact on healthcare and professional culture and investigated how they influenced patients’ timely access to quality care and physicians’ working conditions. Based on these findings, they explored the political economic of healthcare.

In low-income countries the analysed research showed that, through loans and cooperation, multilateral agencies restricted the function of public services to disease control, with subsequent catastrophic reductions in access to care, health de-medicalisation, increased avoidable mortality, and failure to attain the narrow MDGs in Africa.

The pro-market reforms enacted in middle-income countries entailed the purchaser-provider split, privatisation of healthcare pre-financing, and government contracting of health finance management to private insurance companies. To establish the materiality of a cause-and-effect relationship, the authors compared the efficiency of Latin American national health systems according to whether or not they were pro-market and complied with international policy standards.

While pro-market health economists acknowledge that no market can offer equitable access to healthcare without effective regulation and control, the authors showed that both regulation and control were severely constrained in Asia by governance and medical secrecy issues.

In high-income countries they questioned the interest for population health of healthcare insurance companies, whilst comparing access to care and health expenditures in the European Union vs. the U.S., the Netherlands, and Switzerland. They demonstrated that commoditising healthcare increases mortality and suffering amenable to care considerably and carries professional, cultural, and ethical risks for doctors and health professionals. Pro-market policies systems cause health systems inefficiency, inequity in access to care and strain professionals’ ethics.

**Conclusion:**

Policy research methodologies benefit from being inductive, as health services and systems evaluations, and population health studies are prerequisites to challenge official discourse and to explore the historical, economic, sociocultural, and political determinants of public policies.

## Background

Since the 1980s, markets have turned increasingly to intangible goods – health care, education, arts, and justice. Political changes have accompanied the transformation of health systems. After World War II, the WHO was founded to counter the health effects of devastating destruction, but over the last decades its funding by the World Health Assembly dropped to 25% of its budget. Foundations and industrial countries funded the rest, that is, their preferred programmes. The 1978 Alma Ata Declaration establishing the Primary Health Care policy had resulted from able WHO leadership and a growing social movement demanding health for all. One year later, the Selective Primary Health Care movement promoted by the Rockefeller Foundation undermined its foundations. It led the international policy exclusively to support disease control programmes in LMICs and to turn their first-line health services into epidemiological units allegedly because comprehensive primary health care was costly.

After the collapse of the “socialist” camp in 1989, the Washington Consensus, WB, and IMF conditioned low-interest loans on moves to market economy and government withdrawal from health care provision and financing. Since the 2000s, governments in industrialized countries and their private sector set up international disease control programmes called Global Health Initiatives. These were actually epidemiological public-private partnerships that replaced international cooperation in the health sector.

With the Millennium Development Goals (MDGs) and subsequent Sustainable Development Goals (SDGs), the United Nations set quite unambitious global health goals. They assigned donor-driven targets to LMIC governments, that is, controlling a limited number of pathologies, first transmissible and then increasingly chronic ones.

Over this period the authors evaluated pro-market reforms and policies and identified their determinants through the lenses of patients, physicians and health professionals, and taxpayers. Patients are concerned about accessibility to healthcare services and the price and quality of care. Physicians’ interests are, or should also be, their problem-solving capacity, professional freedom, intellectual progress, medical ethics, work environment, and income. These were the authors’ yardsticks to assess health systems and conduct policy research. These studies thus covered curative medicine, preventive medical care, and medical education but not the important field of inter-sectoral public health policies.

It all started in 1982, when J.-P. Unger discovered in Boston the Rockefeller Foundation’s long-term strategy to commoditise healthcare financing worldwide. In investigating the health marketisation motives of the “Selective Primary Healthcare” strategy in low- and middle-income countries (LMICs), he interviewed J. Walsh and K. Warren, the authors of a publication released just 1 year after the Alma Ata conference that advocated an alternative to the Primary Healthcare strategy called “selective primary healthcare” [1]. Their message was that the Primary Healthcare Strategy endorsed by the World Health Assembly in Alma Ata in 1978 was unaffordable. Instead, the Rockefeller Foundation promoted a policy that would turn low-income countries’ (LICs) public health systems into structures fit to host disease control programmes – “like Christmas ornaments festooning a Christmas tree” – rather than delivering individual health care. A field experiment in Deschapelles, Haiti [2], was a central piece of evidence supporting this strategy to make LIC health centres in public services mere disease control structures. The scenario pushed by the Rockefeller Foundation eventually came to be in LICs in the 1980s, albeit with major variants.

In 1986, we invalidated the efficiency alibi of this strategy. As an answer to the Rockefeller strategy, an action research project covering 180,000 persons in Kasongo, Congo [3] (then Zaire), enabled us to show that the cost of delivering individual health care and a few disease control and other public health interventions under a single administration could be similar to those of first-line services providing just five disease control programmes, because the former solution made it possible to keep its administration simple [4]. That prompted us to study the economic motives and public health consequences of healthcare insurance commercialisation, healthcare commoditisation, and health service privatisation and to build a case with coordinated studies. This paper meta-analyses the objectives, methods, and results of evaluations and research into market based health systems and policies spanning over 35 years (https://pure.itg.be/en/persons/jeanpierre-unger(92d91a56-f267-4b85-82e7-9e4f8a8cffed).html). Specifically, it aims to make sense of an array of policy studies that all relied on the same medical and public health ethical criteria already formulated in 1972 [5]; and to delineate health policy research standards relevant to physicians, health professionals, and patients’ representatives committed to the human right to health, i.e., the right to access professional care in universal health systems [6].

### Research strategy

On the grounds of the Kasongo experience and aforementioned Walsh and Warren interview, we formulated in 1983 the overarching hypothesis of our decades-long policy research: Pro-market reforms of healthcare financing and management expand the healthcare delivery and disease control market to the detriment of patients, populations, doctors, health professionals, and taxpayers.

To confirm or overturn this hypothesis, we tested four secondary hypotheses and tried to show a *causal relationship* between pro-market policies’ characteristics and the following phenomena:

1. Regarding the access of patients and persons with health risks to professionally delivered healthcare, we tried to verify whether the market tended to allocate individual, “discretional” health care to the rich and public health interventions to the poor, thereby reducing the general population’s access to care significantly.
2. Regarding disease control, we checked whether public health programmes often failed because the market assigned them a vertical structure to be better suited to absorbing medical equipment and pharmaceuticals with public financing.
3. Regarding fiscal justice, we strove to determine whether health markets ran counter to social justice in health as they precluded the efficient and equitable use of taxes in the care sector.
4. About professionals’ ethics and personal development, we aimed to verify if care commoditisation was compatible with the physician’s reliance on professional ethics and investments in medical equipment and pharmaceuticals might antagonise the conditions of doctors’ and teams professional development.

## Main text

This paper meta-analyses the authors’ research evaluating the impact of markets on health care and professional culture and investigating how they influenced patients’ timely access to quality care and physicians’ working conditions. Based on these findings, they explored the political economy of health care. However, there was no early design of a long-term research strategy. They conducted the studies according to opportunities, although some principles were adopted from the start:

### Interdisciplinarity

Testing the above hypotheses required ad hoc, interdisciplinary research methods in order to build a good case for a causal relationship.

### Heterogeneous research setting

The hypotheses had to be tested in a large array of health systems, from low- to high-income countries. To allow generalisations about the healthcare environment, countries and regions would be key policy analysis units.

### Inductive reasoning

Historical studies would be based on public health evaluation of healthcare systems. Interpreting policy decisions critically required previous ex-post demonstration of ill-functioning services.

### Praxeology

**Figure 1:**
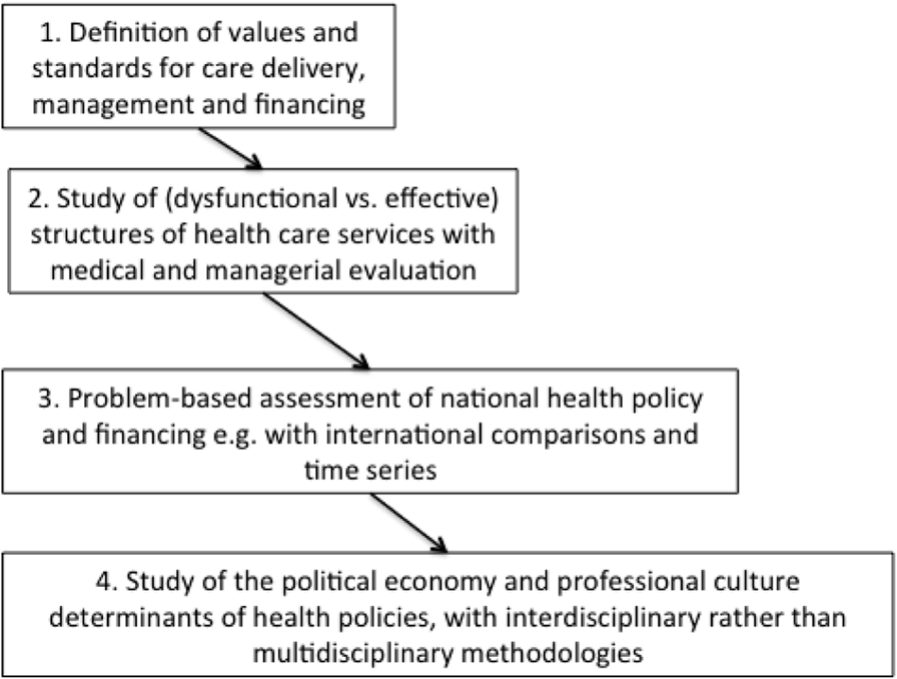
Sequencing the authors’ research on (inter-)national health policies

The authors approached qualitative research in medical care and public health policies by making use of the concept of praxeology that Bourdieu developed and adapted to sociology in his “Outline of a Theory of Practice.” [7] They took this approach because both medicine and public health, like sociology research, are combinations of practice and theory [8]. They believed that the failure to connect them was a frequent weakness of contemporary medical and public health research. An important aspect of praxeology is inductive reasoning. It builds on and evaluates propositions that are abstractions of observations of individual instances of members of the same class. In this regard, the policy evaluations were problem-based and relied on paradoxical observations of care delivery and health service management. They were the raw material of the research and prerequisites for assessing health systems and policies and then exploring the social, political, and economic determinants of faulty ones. Figure 1 depicts the inductive chain generally used in these policy analyses.

### Deconstruction of the policy discourse

Deconstruction is a form of critical analysis concerned with the relationship between text and meaning. Jacques Derrida’s 1967 work on grammatology introduced the majority of its influential concepts. The authors set out to deconstruct public policies with qualitative, interpretative research and nested probabilistic studies. Their goal in this respect was to verify the evidence sustaining pro-market reforms in LMIC and high-income country (HIC) settings; based on these findings, expose their practical, political economy rationale; and then tentatively deconstruct the pro-market discourse of multilateral agencies and commercial organisations. Case studies of national healthcare policies and disease control programmes would provide the material required to analyse international policies and national health sector reforms [9].

### Explicit research values

The authors made explicit their ethical values of social justice and medical professionalism because research methodology, policy evaluation, and interpretation depend on social, economic, and professional standards. These values, published elsewhere, were conceived of for healthcare delivery, management, planning, financing, and disease control. In particular, the authors relied on three healthcare standards with policy implications formulated in 1971, namely, holistic (biopsychosocial and patient-centred), continuous, and integrated care [5]. In Belgium, they served as an ideology to cement alliances of professionals concerned about quality and equitable access to care for more than 40 years [10, 11]. The authors also relied on another key standard of medical practice, the Hippocratic “self-effacement” tenet (“Into whatsoever houses I enter, I will enter to help the sick, and I will abstain from all intentional wrong-doing and harm”) that is expected to deter physicians from making self-interested clinical decisions and maximising their profits with ad hoc clinical decisions, i.e., practising commercial medicine.

## Results

### Evaluating disease control programmes, the hub of international and national health policies in LICs

By 2015, Africa still had not attained the modest MDGs in health. In 2007, we reviewed the grey literature issued by the main multilateral agencies active in the LIC health sector. Under the aegis of the MDGs, disease control was the conceptual and operational hub of health system reform in LICs. Our review revealed that over the preceding 25 years, virtually all the multilateral agencies active in the health sector had adopted policies restricting the function of LICs’ public services to disease control, thereby allocating individual healthcare delivery to commercial services (and private, non-profit facilities where they existed) [12].

To convince physicians and policy makers in LICs to adhere to sectoral reforms and to replace individual care delivery by disease control in public services, the Bretton Wood agencies attached conditions to their loans and projects and financed a host of local experts to produce the “scientific” justifications of this policy.

**Table:** 

In Sub-Saharan Africa and the Andean countries, the multilateral agencies advocated allocating public budgets to the most efficient disease programmes, chosen on the basis of Disability-adjusted Life Years (DALYs) and Quality-adjusted Life Years (QALYs). Alleged efficiency gains were used to justify to doctors and nurses the idea of offloading individual care delivery from public services’ duties. However, in practice, DALYs and QALYs were rarely used to define disease control priorities. Planning could not have been their motive, because the underlying methodology entailed extensive data collection, was flawed by major inconsistencies (for instance, drawing on efficiency in allocation instead of productive efficiency) [13], and had probably never been intended to be translated into actual policy practice [14]. While the availability of funds for Global Health Initiatives (GHIs), rather than DALYs and QALYs, appeared to be the key trigger of new international disease control programmes, these indicators ranked high in the theoretical justifications of LIC policies, thus revealing the importance of ideology in public health science and the role of science in health systems’ reproducibility.	

For LIC populations, the avoidable mortality, suffering, and anxiety that followed the loss of access to individual care proved immense. In Africa, virtually none of the MDGs were attained, regardless of their limited scope, precisely because in failing to deliver individual healthcare, African public services could no longer implement disease control initiatives satisfactorily.

To explain why a huge financial effort (AIDS control funds, for instance, were multiplied twentyfold between 1997 and 2007) could not achieve the MDGs in Africa, the authors

- showed mathematically that successful disease control programmes required health facilities to be used by patients with various symptoms, as they represented the pool of users that these programmes needed for early case detection and follow-up [15].
- studied the mechanisms whereby integrated disease control interventions hampered access to care in the services in which they were integrated and so undermined public services. Although a few AIDS and under-five programmes had been known to deliver bio-psychosocial care, disease control programmes in Africa have reduced the problem-solving capacities of health services; shrunk the professional identity and skills of physicians and nurses; reduced access to drugs to those managed by Global Health Initiatives; and limited in-service training to collective care delivery [16].
- showed this to be a “catch 22” situation, with disease control programmes drastically reducing the number of users in the (public service) facilities where such programmes were implemented [17].
- analysed the evidence of pro-market policies for other characteristics, such as equitable access to quality health care; mismatch of commercial healthcare delivery with medical ethics [18]; the inability of public services focusing on disease control to respond to people’s demands for individual care, thus preventing community participation; and undue restrictions on professional autonomy in health services designed as “machine bureaucracies.” [19]

The authors concluded that Hypothesis 2 was plausible because of the following:

- Disease control-based reforms strained access to care in LICs without achieving their alleged epidemiological goals.
- Replacing individual health care by disease control interventions in LIC public services could be the real motive of the related (inter-)national policies. This was because these reforms practically, albeit tacitly, ushered in a situation in which competition between public and private providers in delivering individual care was made impossible. Multinationals linked to charities that were focusing LMIC public services on disease control took advantage of the disappearance of publicly delivered health care to sell medical care to LMIC middle and upper classes without having to face public sector competition. International disease control programmes not only permitted the use of cooperation funds to purchase drugs and medical equipment manufactured by HIC industries, so fomenting aid-dependent pharmaceutical markets in LICs, but were also structured to foster the healthcare market in urban settings.

### How do health-financing markets perform in middle-income countries? Comparing Latin American national healthcare policies and evaluating healthcare regulation in Asia

In MICs, pro-market health system reforms focused on national health care financing. Starting in Chile in the 1980s (under a military government) and in Colombia in 1993 (under an authoritarian government), the privatisation of health financing in Latin America occurred in virtually every country, even those with “socialist” governments. The two exceptions that did not undergo market reforms, Costa Rica [20] and Cuba [21], were performance outliers. However, the reform scenarios and organisation of health systems were not identical across the continent. Schematically, Insurance companies made profits whilst managing government funds, capturing the health expenditures of the healthy and wealthy middle class, and employing or contracting physicians. The political economics of health sector reforms in MICs consisted of variable combinations of

- under-financing public services;
- unduly favouring investments in public services over their recurrent operating costs;
- putting the physicians working for publicly-oriented institutions under economic and workload stress;
- separating purchasers and providers by law so as to create a niche for commercial insurance banks;
- allowing commercial entities to manage public funds and possibly making this scheme mandatory;
- privatising public hospitals or imposing commercial competition rules on them (the so-called “management property split”) and on contracted, self-employed physicians, too;
- stimulating private financing of public hospitals (“private finance initiatives”);
- limiting public services’ activities to unprofitable care, e.g., for the poor (Medicaid) and the elderly (Medicare) in HICs, and to disease control programmes in LMICs; and
- liberalising investments in health care under the aegis of international trade treaties.

Given the many cultural and political similarities across Latin American countries, their health systems offered a good setting to explore strategic variants of care commoditisation. The authors assessed primarily the effects of pro-market reforms in Latin America by comparing the performance of systems abiding by international (World Bank, International Monetary Fund, Inter-American Development Bank, etc.) health policies with those that did not [9]. They thus studied the histories and functioning of some national health systems and the impact of financing options on their management, care quality, and access to care. To study health systems’ productivity, they relied on aggregated production data, population-based care accessibility and continuity rates and ratios; direct observations in healthcare services and administration; and interviews of patients, physicians, health professionals, policy makers, and public health experts.

They studied the health care and outcomes of large-scale, nationwide, in vivo experiments of care commoditisation. The ones they studied did not show any benefits for patients, physicians, health professionals, and/or public finances:

- Colombia, which had been a good student, by international standards, since 1993, had a deplorable health record [22]. In our interviews we studied and compared the barriers to access to care erected in Colombia by a managed competition model with the barriers in north-eastern Brazil, where public services were severely under-financed [23, 24]. As expected, both had very poor results.
- In 2006, Chile’s public services [25], which had survived the dictatorship, covered 84% of the population with half of the country’s health expenditure. However, with just 50% of the country’s health expenditures, the public services managed to make the country a positive outlier in Latin America on many health indicators. The technical challenge of this study was to relate health system features to indicators of output (utilisation and coverage rates, for instance) and outcome (maternal mortality, for instance).
- Finally, in 2001, Costa Rica, with its publicly-oriented healthcare services and financing, had about the same demographic and epidemiological features as the United States, although it spent nine times less per capita on health than the U.S. [20]

To fuel the legal and institutional dynamics of health insurance privatisation, the WHO and other UN agencies promoted a strategy called “Universal Health Coverage” (UHC) [26], that is, universal access to health insurance. Its pro-market discourse endorsed the idea that only insured populations could access health care [27], despite evidence that expanding insurance coverage might reduce service utilisation, e.g., when public-private insurance mixes were supposed to achieve universal coverage of health risks [28–30] and evidence of the superior effectiveness, fairness, and efficiency of Latin American off-market health systems [20, 21].

The findings of these international comparisons led the authors to question the UHC strategy as a way to secure universal access to care. This was not only because public-private mixes in healthcare financing give rise to severe inefficiency in health systems, but because access to care was shown to be highly dependent on non-financial factors (geographical and psychological accessibility of health services, for instance) [31] otherwise neglected by the UHC strategy and possibly even undermined by it. In the absence of performance-based evidence supporting health-financing marketisation, the hypothesised centrality of an economic agenda in Latin American health reforms became plausible.

In sum, these comparisons of the Chilean, Colombian, Costa Rican, and Brazilian health systems and historical studies of Bolivia and Ecuador support Hypothesis 1 regarding the negative impact of pro-market policies on access to care and quality of care and Hypothesis 3 regarding fiscal injustice and inefficient use of public funds by commercial health services and insurance companies.

In addition, the authors’ studies of Asian health systems showed that the health care market was structurally flawed by the impossibility of regulating and controlling the activities of the private but also public health care sector in MICs properly. Whilst the Rockefeller Foundation had tacitly admitted that without regulation and control, privatising health services could not produce equitable access to care [32], the authors showed through their observations in nine (maternal health) case studies of regulations in China, India, and Vietnam [33] and theoretical discussion [34] that regulation and control of for-profit care delivery were most likely to be ineffectual in the MIC care sector.

In Vietnam, for instance, sex-selective abortion was responsible for a serious gender imbalance in spite of a decade of State regulation and control. Although a regulation against the practice had been passed in 2003 and implemented since 2006, regional disparities in gender-specific birth rates increased between 2006 and 2011. As a “critical incident”, the number of ultrasound violations detected in 2011 had been 1 positive out of 83,192 controls done in the health districts under study. And in 2016, the gender ratio still was 112 females/100 males in Vietnam. Against a background of strong social demand for sex-selective abortion in the middle class, selective abortions remained undetected in spite of the regulation and inspections because of the policy-makers’ failure to allocate sufficient resources to this exercise, weak governance, medical secrecy, conflicts of interests, dual physicians’ employment (in public and private healthcare services), the opacity of the medical market, and difficulties specifying contingency in clinical situations [33].

This set of nine studies in China, India, and Vietnam thus supports the plausibility of Hypothesis 4, as they confirm the vulnerability of medical ethics to care commoditisation policies when regulation and control of medical practice are ineffectual, which actually they are because of the socio-political and technical characteristics of middle income countries.

### Assessing the impact of health markets on access to care in Europe

At the end of World War II, unionised blue-collar workers imposed social protection schemes in health. In a bipolar world, the workers’ organisations took advantage of progressive ideologies that were gathering strong followings in Europe. Whilst the weakened employers’ organisations prevailed upon the Social Democrats and Social Christians to join them in the anti-Communist struggle, they conceded the pillarization of European States. Workers’ trade unions, mutual societies, and political parties entered the parliaments (as was the case before Word War II), but also the State’s executive branches, judiciary, and social and health services, education, the police, and the army. That is how workers’ representatives limited the impact of corruption in State constituencies, i.e., preventing those who had the will and resources from buying the State’s policy and administrative decisions. They locked the sustainability of social security into government structures and secured access to professional health care in universal health systems as a human right. Admittedly, the users of healthcare services paid for this State pillarization with a dose of nepotism and its consequences. Still, European States had acquired key democratic features. Heated negotiations between representatives of social classes with opposing interests produced sectoral priorities within the overarching framework of national health budgets. In Belgium, for instance, this debate was institutionalised in the national social security organisation.^1^

The macroeconomic result of this pillarization of the State can be seen in two inversely proportional numbers that show the importance of risk-pooling and solidarity in European health care financing, namely:

- a government share in total health spending that long exceeded 80% and
- total health expenditures that were high enough (about US$4000 per capita in 2014, of which approximately 10% was for the commercial sector) to make the publicly-oriented healthcare services^2^ effective but sufficiently modest (10% of European GDP versus 17.1% of U.S. GDP in 2014) to *favour* economic growth outside the health sector.

That is how employees’ and employers’ taxes and social contributions made it possible to limit household expenditures on health care whilst securing one of the best geographically, financially, psychologically, and technically accessible forms of professional health care. Importantly, these schemes gave physicians sufficient professional autonomy. Access to professional care was equitable thanks to cost-redistributing, non-profit, non-actuarial health care financing and a sufficiently large proportion of *non-material* investments in the health sector.

With government social security schemes that included fairly comprehensive universal health insurance, Europeans enjoyed a high degree of social protection from 1945 to 1989 in Eastern Europe, until the 2008 financial crisis in Southern Europe, and even later in other countries.

Unfortunately, the institutional pillarization did not prevail at the European Union and Commission level. Rather, European politicians, civil servants, and political parties were the targets of more than 30,000 commercial lobbyists (1.4 per European Commission (EC) civil servant) [35] working to foster the interests of the international insurance banks that were investing in health, amongst other things. In contradiction to the provisions of the Treaty of Rome [36], the EC intervened in the Member States’ health care systems by negotiating international trade treaties involving investments in health care that could make healthcare management and medical practice subject to a commercial rationale. In addition, the 3% budget deficit rule that the Maastricht Treaty imposed on Member States gave political parties an opportunity and a plausible reason to cut public expenditures on health until healthcare financing would be sufficiently privatised, as the WB and IMF had done earlier in Latin America.

Public expenditure on health care was severely constrained but once health laws and regulations had been modified, as shown by the history of Dutch, Swiss, and Colombian health systems, insurance banks strove to maximise public and private expenditures on health care and governments found the needed resources through inter-branch arbitration.

In the U.S., where the health market was mature, the wealthy faced more problems accessing health care than the poor in most OECD countries, whilst the U.S. government alone spent more on health per capita than the total (public and private) per capita spending of most European countries [37]. Nevertheless, over the last 10 years, the number of uninsured Americans varied between 35 and 50 million. Many more were poorly insured. If the U.S. insurance coverage rate were applied to Europe, the number of uninsured Europeans would reach about 75 million. If the European ratio of mortality amenable to care became that of the United States, avoidable mortality would increase by up to 100,000 deaths per year.

In Latin American countries, the same financial structure yielded the same health effects as in the U.S. but, admittedly, not in the Netherlands and Switzerland. The sustainable performance of these two health systems is central to policy debates in Europe and, expectedly, insurance banks praise their functioning, except for one small detail: Since health care financing has been marketed (respectively in 1996 and 2006), the Swiss and Dutch health expenditures have skyrocketed [38].

What are the reasons to believe that health insurance markets are environments hostile to the universal right to care? The authors evaluated [39] the performances of the U.S., the Netherlands, and Switzerland, three industrial nations that pursued market-based financing models, with a focus on equity in access to care, care quality, health status, and efficiency. They then assessed the consistency of their findings with those of various research teams. Using secondary data obtained from a semi-structured review of articles from 2000 to 2017, inter alia, they discussed the hypothesis that commercial health care insurance was detrimental to access to professional health care and population health status.

The findings can be summarised as follows:

- In 2010, poor Americans had twice the unmet care needs of Americans with above-average incomes and ten times more than the UK poor. The unmet care needs of the rich in the U.S. exceeded those of the poor in several industrial countries [40]. The number of Dutchmen and -women experiencing financial obstacles to health care quadrupled between 2007 and 2013 [41]. Switzerland ranked second worst in a 2016 survey of 11 countries, just ahead of the USA, with 22% of Swiss adults likely to skip needed care [42].
- The most negative impacts of “managed care” on care quality were its tight constraints on physicians’ professional autonomy, large reliance on the physicians’ material motivation, the fragmentation of health services, and a tendency to apply evidence-based medicine too rigidly. In requiring strict application of clinical protocols, commercially managed care was less likely to be favourable to care quality than systems giving physicians sufficient freedom to rely on professional decision-making and medical ethics.
- The prevalence of burnout amongst MDs made medical practice the riskiest occupation in the United States and one of the riskiest occupations in Europe [43]. This burnout was not related to insufficient income but to excessive workloads and to perceiving existential threats to their professional identity, ethics, and autonomy in the way health care was organised. This observation supports Hypothesis 4 because these psychological and professional status threats actually result from the commoditisation of care [44].
- Countries with a commercial insurance monopoly generally remained above the maternal, infant, and neonatal mortality rates v. the health-spending regression line [45]. And the growth rates of health expenditure were the highest in the U.S. and Switzerland, with the Netherlands not far behind [46].
- Controlling for the impact of the obesity confounding factor, these studies reveal that the industrialisation of care contributes to the comparatively poor performance of the U.S., Dutch, and Swiss health systems, with the Dutch first-line services being an exception made possible by the GPs’ medical culture and the low cost to patient.
- International trade treaties may further worsen the mortality rates of cardiovascular and cerebrovascular conditions, diabetes, and cancers in Europe, since they favour the food industry’s market penetration [47].

These findings admittedly conflict with recent influential health system rankings, perhaps because of the ways their health indicators are constructed and a bias towards assessing first-line healthcare services.

In conclusion, the comparison of US, Dutch, and Swiss health systems with the others in Europe supports the validity of Hypotheses 1 and 3. The most inefficient system is where the insurance market has achieved its maximal development, that is, in the U.S. In general, healthcare expenditures rose faster where health insurance was commoditised. The Netherlands and Switzerland reveal that increasing expenditure on health care enables health systems based on commercial insurance to maintain decent access to professionally-delivered health care for a few years.

The sizeably better, much more equitable access to health care in Western Europe (and its demographic and epidemiological superiority over the U.S.) and its much lower cost is generally explained by redistributive laws and regulations (tax-based or mandatory social security) channelled through health care public services or mutual societies that permit solidarity in health care financing.

The analysis of the U.S. health system’s disappointing performance reveals that actuarial management of health finances and the commercial management of health services are responsible for deficient accessibility to care and services. In particular, actuarial management of health care reduces risk pooling and solidarity in health financing between men and women, the young and the elderly, the sick and the well, high and low risks, and rich and poor.

### Methodological lessons for descriptive, policy studies

- Identifying health services productivity shortfalls and dysfunctional structures

The authors tried to provide patients’ and physicians’ organisations with the evidence and clues about policies from the angle of the human right to care and professional endeavour. Their research assessed the influence of policies on health services’ productivity in defined historical contexts from various standpoints: those of patients (e.g., care quality and accessibility); physicians (e.g., continuing medical education and teamwork); taxpayers (efficiency and equity in use of public monies); and public health specialists (health care and disease control management).

From an inductive study perspective, documenting health services’ structural and functional deficiencies provided the raw material for assessing health systems and possibly challenging policy decisions and official discourse.

To gauge the quality of health care, the authors used medical knowledge to observe clinical practice (sometimes as mock patients) [48]. For instance, to assess the impact of managed care techniques on care quality in Costa Rica, they sat in on consultations. The research hypotheses had been formulated by the Limon region’s GPs, who suggested that there was a relationship between managed care (*compromisos de gestión*) and the lack of time available for interpersonal communication and deficient care accessibility [49]. In addition, they collected data on disease-specific indicators to explore the extent to which managed care techniques were responsible for decreasing care quality and data reliability.

To assess care accessibility, they often used the services’ routine production data, with indicators such as population-based utilisation rates of curative care in first-line services and hospital admission rates [31], referral completion rates, and preventive (vaccination, antenatal clinics, etc.) coverage rates, and then they validated them by triangulation when possible. As a proxy for the financial accessibility of health care, they used “catastrophic health expenditures.” [50] Routine data proved cheaper, readily available, and a good reflection of the services’ operations in large geographical areas, but the method had limits even when it was combined with data triangulation and controls:

- In Colombia, semi-structured interviews of patients and professionals proved indispensable to gauge care accessibility [51–53] because networks of “sentinel physicians” were not organised to collect service utilisation and epidemiological data; population-based statistics were not available and the denominators would have consisted of populations affiliated with a myriad of health insurers and care providers; and private insurance companies were reluctant to provide data that could undermine their reputations.
- The routine data were sometimes biased, such as in the case of a state administration in charge of determining regional maternal mortality rates in Asia. Aside from the technical difficulties of establishing the maternal mortality rate (MMR), middle line managers were likely to be penalised when this indicator was too high but also too low, because in the latter case the administration did not trust the data’s validity [33]. Hence a regression to the mean …

In general, the authors relied on output indicators rather than on population outcome. However, two demographic indicators proved particularly interesting for critical assessment of healthcare systems:

- The Maternal Mortality Rate (MMR) reflects access to the entire healthcare system pyramid [54], particularly in LMICs [55] and probably in any situation where it exceeds 40 per thousand. This is in contrast to the Infant Mortality Rate (IMR), which in LICs often mirrors low-cost interventions that may reduce access to care (such as immunisation campaigns) [56] and biomedical/sociocultural health determinants (such as the availability of food and clean water and women’s education, respectively). Since the lower the per capita GDP, the cheaper and less reliable the demographic indicators used [57], the authors retained in practice only the gross differences when comparing the health systems’ performances in terms of MMR. In 2010, for instance, Moldova, the poorest country in Europe, had the same MMR as the U.S., despite spending 1/20 as much on health per capita.
- In HICs, life expectancy and population mortality rates mirror obesity-associated pathologies but, just as importantly, access to quality health care. Up to 80% of premature deaths in Poland were explained by unsatisfactory access to health care [58]. According to Kruk and co-workers, 15.6 million excess deaths from 61 conditions occurred in LMIC in 2016. This research compared case fatality between each LMIC with corresponding numbers from 23 high-income reference countries with strong health systems. After excluding deaths that were preventable by public health measures, the authors found that 55% of excess deaths were amenable to health care and could be put down to either the receipt of poor-quality care or the non-utilisation of health care [59].

To evaluate health systems by the design and performance of their disease control programmes, the authors relied on two models:

1. An all-purpose disease control model (“*vertical analysis”*), designed by P. Mercenier [60] to provide standards for the design of disease-specific control programmes. It was based on the systemic representation of the disease-specific syndromes and vector development stages and biomedical and socio-cultural interventions to interrupt the disease chain in the field, from aetiology to patient death.
2. M. Piot’s model [61] to assess care continuity for any defined disease. It establishes the disease-specific cure rate as the product of coefficients measuring detection, diagnosis, and treatment activities. As the model reveals the health system characteristics needed to secure, say, early detection and care continuity, they used it to contrast the performances of public and private sectors in tuberculosis control in India [62] and to evaluate malaria control programmes in Mali and Sub-Saharan Africa in general [15].

Once health system productivity had been studied, the authors analysed the organisation of health services and systems. For this they relied on managerial models and standards specific to

- publicly-minded care management (e.g., concerned with access to professional health care, professional autonomy and well-being, professional ethics, and public health) [63];
- the systemic management of hospital(s) and first-line facilities networks [18]; and
- “divisionalised adhocracy”, an organisation pattern that favours knowledge management and teamwork [19] and is suited to systems whose end-line producers are highly skilled and sufficiently autonomous professionals (as are physicians) rather than workers and technicians, as assumed by the classic generic management theories.

b. Studies of health financing and systems characteristics that cause low services productivity

Health system case studies and the existence of large databases in the health sector provided the opportunity to single out natural, quasi-experimental study designs – time series and non-equivalent comparisons – to contrast health systems with and without or before and after pro-market health reforms:

- For non-equivalent groups (countries, regions, etc.), the authors compared the performances of national/regional health systems in Latin America compliant with the international policy standards with those of “disobedient” ones [9] and established a typology of reforms.
- With time series, we showed long-standing, substandard performances in the quality, accessibility, and financing of health care (for instance, after the privatisation of health insurance in Colombia).

Time series of health services’ routine data also proved useful to reveal contradictory interactions of health activities in populations. For instance, in the late 1980s, the utilisation of medical consultations decreased steadily in Senegal whilst immunisation campaigns were implemented in health care services [64]. The challenge of the study consisted in demonstrating a causal relationship between these campaigns and the subsequent sustained deterioration of care accessibility in public services.

c. Beyond substandard care performances: political economics

Inductive research made it possible to deconstruct official self-apologetic discourses. The authors were then able to seek the real motives for ill-conceived policies whose results belied the stated objectives. Their entry point in the complex socio-cultural and political determinants of health policies was political economics because of the huge weight of health expenditures in the global economy (up to 17% of U.S. GDP and 11.3% of Germany’s GDP) and the political leverage acquired by the economic players. The economic determinism of health care policies was so powerful that these players did not even need to be coordinated to gear health systems towards care markets [65].

From corruption [66], political leverage, and lobbying to trade, it takes time for relationships between commercial organisations and public institutions to result in health systems’ structures and new professional practice. Some studies thus adopted an historical viewpoint [12, 65, 67, 68] to probe the care commoditisation mechanisms. Even in non-profit organisations, the main determinant of poor healthcare accessibility proved to be the business mission of health financing, management, and medical practice.

However, correlations between events, sequences, sociological observations, and relationships between historical times enabled us to identify professional, cultural, and geostrategic determinants of health policies alongside economic ones. The prevailing order was reflected in professional culture thanks to education, information, scientific ideology, and advertising. The resulting personal characteristics, identity, and knowledge of physicians and professionals were the conditions of health systems’ reproducibility. Bourdieu calls these internal features “habitus,” i.e., ways of doing and being, and “representations”.

## Conclusion

Since 1985, the trend has been towards the privatisation of health financing, public subsidies for private health care providers, commercial management of health services, and for-profit medical practice, in spite of the wealth of evidence pointing to the risks of large-scale mortality and morbidity and threats to professional ethics associated with the commoditisation of care.

Governments and multilateral agencies ought to be held accountable when health policies cause avoidable mortality and suffering and thus human rights violations, or at least “be shamed”, as Sir Michael Marmot once said. Therefore, with States being fields “structured according to oppositions linked to specific forms of capital” [69], health system and policy research should not so much address the knowledge needs of policy makers directly as those of physicians, socially-minded professionals, and patients’ organisations that could leverage them. Political indictments on the impact of health policies require these organisations to access the relevant scientific and professional information in order to question and challenge public policies in the health sector.

The studies analysed here stemmed from the human right to access professional care in universal health systems and the knowledge they produced was directed at physicians, health professionals, and patients’ organisations sharing moral values and interested in lobbying health policies. The present meta-analysis sheds light on the requirements of this type of research:

- Inductive, multidisciplinary policy research is time-consuming but often a condition to study health policies independently:
  International health policies assessments benefit from analysing national healthcare policies and disease control programmes.
  National health policies should be studied with political economy and medical concepts, and through the lenses of political science and history, but importantly on the grounds of health systems and services productivity assessment.
  Medical concepts, public health models, and indicators of professional care delivery and non-profit health management make it possible to evaluate health systems from a professionally- and socially-driven, problem-based perspective.
  Health systems and policy researchers need scientific and professional knowledge. Academics should engage in medical, managerial, and policy-making work alongside their research and teaching activities. Therefore, medical and public health schools should learn to assess the academic’s professional proficiency and ability to derive validated theory from their practice.
- Professional ethics should be a criterion for evaluating care quality:
  Although values are an obstacle to Weberian axiological neutrality in medical, public health, and education policy studies, they are indispensable to assess care quality, health services, and healthcare systems. From a phenomenological perspective, they ought to be made available to the reader.
  Health systems have evolved rapidly over the last three decades. Long-term reliance on the same set of explicit ethical and technical criteria applicable to medical practice and health services organisation is what allows valid conclusions to be drawn from time series and comparative or historical studies of health systems that belong to different eras.
- Such studies ought not to be only descriptive and critical but also designed as proposals to improve health systems and policies. Those analysed here reveal many nationwide experiences to improve access to professional care. Some countries (Costa Rica, Cuba, Spain, Sri Lanka, Thailand, and Italy), states (e.g.*,* Kerala), regions or cities (e.g. Rosario, Argentina), and health systems (Chilean public services) acquired collective knowledge to develop non-commercial care delivery and promote ethical, medical practice. There is no doubt that decades of neoliberal policy have compromised their professional achievements, to the point that they are often no longer perceptible.
- Medical journals ought to be devoted to professional practice and not only to science, and be independent and publicly financed. Given the undeclared conflict of interest created by the presence of insurance banks in the shareholding of top impact-factor medical journals, physicians’ and patients’ organisations should lobby public universities to stop relying on the researcher’s bibliometrics and the impact factor to decide on scientists’ careers.

The hypothesis that the authors formulated in 1983 can reasonably be accepted. Health markets most likely undermine patients’ health, physicians and professionals’ status and morale, and taxpayers’ interests. The key function of health sector reforms is not public health but economic: they aim to privatise the profitable part of health care financing; maximise the return on health care with commercial healthcare management of services and for-profit care delivery; prevent public services from being involved in a competition with the private sector for health care delivery, management, and financing; and open markets in LMICs with public aid funds to medical and pharmaceutical goods preferably manufactured in industrialised countries.

The studies analysed here show physicians and their organisations that commercial healthcare financing is incompatible with ethical, medical practice because, with or without vertical integration (in HMOs or PPOs), whether through contracts or wages, it imposes the goal of maximising shareholders’ profits on physicians and health professionals, whereas this commercial mission goes against the grain of Hippocratic ethics.

To patients’ organisations, the studies analysed here prove worldwide that care commercialisation prevents solidarity in healthcare financing and obstructs equal access to care. Markets segment health systems, they foment competition between physicians, whilst cooperation among them is essential to peoples’ health [13]. Moreover, they use public expenditure on healthcare inefficiently.

This research thus opens avenues for joint political action by patients’ and physicians’ organisations to defend and promote social protection in health because it shows that both doctors and patients benefit from professional care delivery and publicly-oriented care financing and management; the major contemporary threat to care accessibility and quality, namely, the privatisation of health care financing, also jeopardises the physicians’ autonomy, ethics, and incomes.

Finally, this research shows that competition prevails between not only commercial entities but also sectors. The interests of insurance banks investing in health and those of all the other economic actors are contradictory: Inter-country comparisons of total health expenditures reveal that the commodisation of care is accompanied by broad inter-sectoral, macro-economic redistribution. Economic agents that do not invest in health insurances would do better to learn from this.

## Data Availability

Data sharing is not applicable to this article as no datasets were generated or analysed during the current study.

## Abbreviations

DALY: Disability-adjusted life years
EC: European Commission
GHI: Global health initiatives
GP: General practitioner
HIC: High income countries
IMR: Infant mortality rate
LMIC: Low and middle income countries
MD: Medical doctor
MDG: Millennium development goals
MMR: Maternal mortality rate
QALY: Quality-adjusted life years
SDG: Sustainable development goals

## Footnotes

1 Whilst these schemes had been forced upon employers, they unexpectedly proved to be highly favourable to economic growth.

2 Referring to health services and systems, we use the terms “publicly oriented”, “publicly minded”, “socially driven”, “non commercial”, and “not for profit” interchangeably

## References

[1] Walsh JA, Warren KS (1979). Selective primary healthcare: an interim strategy for disease control in developing countries. N Engl J Med.

[2] Berggren WL, Ewbank D, Berggren G (1981). Reduction of mortality in rural Haiti through a primary-health-care program. N Engl J Med.

[3] The Kasongo project (1981). Annales belges de médecine tropicale.

[4] Unger JP, Killingsworth JR (1986). Selective primary healthcare: methods and results. Soc Sci Med.

[5] G.E.R.M (1971). Pour une politique de la santé.

[6] Unger JP, Morales I, De Paepe P, Roland M. Medical heuristics and action-research. Professionalism versus science. Forthcoming as part of BMC Health Services Research Volume 20 Supplement 2, 2020: “*The Physician and Professionalism Today: Challenges to and strategies for ethical professional medical practice*." The full contents of the supplement are available online at https://bmchealthservres.biomedcentral.com/articles/supplements/volume-20-supplement-2.

[7] Bourdieu P (2000). Esquisse d'une théorie de la pratique.

[8] Unger JP, De Paepe P, Van Dessel P, Stolkiner A (2011). The production of critical theories in Health Systems Research and Education. An epistemological approach to emancipating public research and education from private interests. Health Cult Soc.

[9] International Health and Aid Policies. Unger J.P., De Paepe P., Sen S. & Soors W., editors. Cambridge University Press; 2010. Available: http://www.cambridge.org/us/catalogue/catalogue.asp?isbn=9780521174268. Accessed 13 Sept 2020.

[10] http://www.maisonmedicale.org. Accessed 13 Sept 2020.

[11] http://www.sante-solidarite.be. Accessed 13 Sept 2020.

[12] De Paepe P, Soors W, Unger JP (2007). International aid policy: public disease control and private curative care?. Cad Saude Publica.

[13] Segall M (2003). District health systems in a neoliberal world: a review of five keypolicy areas. Int J Health Plann Manag.

[14] Van der Stuyft P, Unger JP (2000). Improving the performance of health systems: the world health report as go-between for scientific evidence and ideological discourse. Tropical Med Int Health.

[15] Unger JP, d’Alessandro U, De Paepe P, Green A (2006). Can malaria be controlled where basic health services are not used?. Tropical Med Int Health.

[16] Unger JP, De Paepe P, Green A (2003). A code of best practice for disease control programs to avoid damaging healthcare services in developing countries. Int J Health Plann Manag.

[17] Unger JP, De Paepe P, Ghilbert P, Soors W, Green A (2006). Disintegrated care: the Achilles heel of international health policies. In low and middle income countries. Int J Integr Care.

[18] Unger JP, De Paepe P, Ghilbert P, Soors W, Green A (2006). Integrated care: a fresh perspective for international health policies in low and middle-income countries. Int J Integr Care.

[19] Unger JP, Macq J, Bredo F, Boelaert M (2000). Through Mintzberg's glasses: a fresh look at the organisation of ministries of health. Bull World Health Organ.

[20] Unger JP, De Paepe P, Buitrón R, Soors W (2008). Achievements of a heterodox health policy. Am J Public Health.

[21] De Vos P, De Ceukelaire W, Van der Stuyft P (2006). Colombia and Cuba, contrasting models in Latin America’s health sector reform. Tropical Med Int Health.

[22] De Groote T, De Paepe P, Unger JP (2005). Colombia: in vivo test of health sector privatisation in the developing world. Int J Health Serv.

[23] Vargas I, Vázquez ML, Mogollón-Perez AS, Unger JP (2010). Barriers of access to care in a managed competition model: lessons from Colombia. BMC Health Serv Res.

[24] Garcia-Subirats I, Vargas I, Mogollón-Perez AS, De Paepe P, Ferreira da Silva MR, Unger JP (2014). Inequities in access to healthcare in different health systems. A study in municipalities of central Colombia and north-eastern Brazil. Int J Equity Health.

[25] Unger JP, De Paepe P, Arteaga HO, Solimano CG (2008). Chile’s neoliberal health reform: an assessment and a critique. PLoS Med.

[26] http://www.bmg.bund.de/fileadmin/redaktion/pdf_who/Information_Ministerial_Conference_.pdf. Accessed 22 Apr 2016.

[27] https://oi-files-d8-prod.s3.eu-west-2.amazonaws.com/s3fs-public/file_attachments/bp176-universal-health-coverage-091013-summ-en__1.pdf. Accessed 13 Sept 2020.

[28] Andoh-Adjei FX (2010). Assessing the performance of district mutual health insurance schemes in Ghana. International course in health development 46;2009/2010.

[29] Guarnizo-Herreño CC, Agudelo C (2008). Equidad de Género en el Acceso a los Servicios de Salud en Colombia. Rev Salud Pública Colomb.

[30] Consorcio de Investigación Economica y Social (2011). Investigaciones sobre salud.

[31] De Paepe P, Rojas E, Abad L, Van Dessel P, Unger JP, Unger JP, De Paepe P, Sen K, Soors W (2010). Improving access. International health and aid policies; the need for alternatives.

[32] Lagomarsino G, Nachuk S, Singh KS (2009). Public stewardship of private providers in mixed health systems.

[33] Unger J.P., Van Dessel P., Van der Veer C. & Shelmerdine S. Maternal health regulations in Vietnam, India and China. A comparison across case studies and countries. Deliverable 5.1. HESVIC project ‘Health system stewardship and regulation in Vietnam, India and China’. Institute of Tropical Medicine, Antwerp. A project financed by the European Commission. 154 pages. 2012. Available: https://medicinehealth.leeds.ac.uk/downloads/download/122/hesvic_-_comparative_report_d5_1_120713_final. Accessed 13 Sept 2020.

[34] Shuftan C, Unger JP (2011). The Rockefeller Foundation’s public stewardship of private providers in mixed health systems: a point-by-point critique. Soc Med.

[35] The Guardian (2014). 30,000 lobbyists and counting: is Brussels under corporate sway?.

[36] In Article 152 (consolidated, Amsterdam version of the Rome Treaty): “Community action in the field of public health shall fully respect the responsibilities of the Member States for the organisation and delivery of health services and medical care. In particular, measures referred to in paragraph 4(a) shall not affect national provisions on the donation or medical use of organs and blood”.

[37] OECD (2012). Health data set, the Commonwealth Fund, and the 2012 WHO Global Health expenditure database as in Gapminder.

[38] http://www.oecd.org/els/health-systems/health-data.htm. Accessed 25 Mar 2018.

[39] Unger J-P, De Paepe P (2019). Commercial health care financing: the cause of U.S., Dutch, and Swiss health systems inefficiency?. Int J Health Serv.

[40] Unmet care needs due to costs in eleven OECD countries by income group (2010). Commonwealth Fund Health Survey.

[41] Experienced cost-related access problem, 2007 and 2013. The Commonwealth Fund (2014). Interactives and data.

[42] Osborn R., Squires D., Doty M.M., Sarnak D.O. & Schneider E.C. In new survey of eleven countries, US adults still struggle with access to and affordability of healthcare Health Aff. Published online November 16, 2016. 10.1377/hlthaff.2016.1088 27856648

[43] Shanafelt TD, Boone S, Tan L (2012). Burnout and satisfaction with work-life balance among US physicians relative to the general US population. Arch Intern Med.

[44] Unger J-P. Physicians’ burnout (and that of psychologists, nurses, magistrates, researchers, and professors). For a Control Program. Int J Health Serv. 2019; 10.1177/0020731419883525. 10.1177/0020731419883525 PMC7134576 31672078

[45] Gapminder data drawn from the OECD’s 2012 and 2018 health data sets. OECD QWIDS through www.gapminder.org.

[46] OECD Health Statistics. 2018 https://stats.oecd.org/Index.aspx?DataSetCode=SHA. Accessed 5 Nov 2020.

[47] Thow AM, Snowdon W, Labonté R, Gleeson D, Stuckler D, Hattersley L (2015). Will the next generation of preferential trade and investment agreements undermine prevention of non communicable diseases? A prospective policy analysis of the trans Pacific partnership agreement. Health Pol.

[48] Unger J-P, Marchal B, Dugas S, Wuidar MJ, Burdet D, Leemans P, Unger J (2004). Interface flow process audit: using the patient's career as a tracer of quality of care and of system organisation. Int J Integr Care.

[49] Soors W, De Paepe P, Unger JP (2014). Management commitments and primary care: another lesson from Costa Rica for the world?. Int J Health Serv.

[50] Xu K, Evans DB, Kawabata K, Zeramdini, Klavus J, Murray CJL (2003). Household catastrophic health expenditure: a multicountry analysis. Lancet.

[51] Vargas I, Unger JP, Mogollon A, Vazquez ML (2013). Effects of managed care mechanisms on access to healthcare: results from a qualitative study in Colombia. Int J Health Plann Manag.

[52] Garcia-Subirats I, Vargas I, Mogollón AS, De Paepe P, Ferreira da Silva MR, Unger JP, Vázquez ML (2014). Barriers in access to healthcare in countries with different health systems. A cross-sectional study in municipalities of central Colombia and north-eastern Brazil. Soc Sci Med.

[53] Vargas I, Mogollón AS, De Paepe P, Ferreira da Silva MR, Unger JP, Vázquez ML (2015). Do existing mechanisms contribute to improvements in care coordination across levels of care in health services networks? Opinions of the health personnel in Colombia and Brazil. BMC Health Serv Res.

[54] Filippi V, Ronsmans C, Campbell OMR, Graham WJ, Mills A, Borghi J (2006). Maternal health in poor countries: the broader context and a call for action. Lancet.

[55] Unger JP, Van Dessel P, Sen K, De Paepe P (2009). International health policy and stagnating maternal mortality. Is there a causal link?. Reprod Health Matters.

[56] Unger JP (1991). Can intensive campaigns dynamise front line health services ? The evaluation of a vaccination campaign in Thiès Medical District, Senegal. Soc Sci Med.

[57] Hogan MC, Foreman KJ, Naghavi M (2010). Maternal mortality for 181 countries, 1980–2008: a systematic analysis of progress towards millennium development goal 5. Lancet..

[58] Nolte E, Scholz R, Shkolnikov V, McKee M (2002). The contribution of medical care to changing life expectancy in Germany and Poland. Soc Sci Med.

[59] Kruk ME, Gage AD, Joseph NT, Danaei G, García-Saisó S, Salomon JA (2018). Mortality due to low-quality health systems in the universal health coverage era: a systematic analysis of amenable deaths in 137 countries. Lancet..

[60] Unger JP, Criel B, Mercenier P, Van Lerberghe W, de Béthune X (1998). L'approche verticale: une méthodologie d'identification des priorités stratégiques du contrôle des maladies tropicales. Intégration et recherche Antwerp Institute of Tropical Medicine.

[61] Piot MA (1967). A simulation model of case finding and treatment in tuberculosis control programmes.

[62] Unger JP, De Paepe P, Ghilbert P, Zocchi W, Van Dessel P, Qadeer I, Unger JP, De Paepe P, Sen K, Soors W (2010). Privatisation (PPM-DOTS) strategy for tuberculosis control: how evidence-based is it?. International health and aid policies; the need for alternatives.

[63] Unger J-P, Marchal B, Green A (2003). Quality standards for health care delivery and management in publicly-oriented health services. Int J Health Plann Manag.

[64] Unger JP, Mbaye A, Diao M (1991). Can intensive campaigns dynamise front line health services ? The evaluation of a vaccination campaign in Thiès Medical District, Senegal. Soc Sci Med.

[65] De Paepe P, Echeverria RE, Aguilar SE, Unger JP (2012). Ecuador’s silent health reform. Int J Health Serv.

[66] Lewis M. Governance and corruption in public healthcare systems: Center for Global Development. The World Bank; 2006.

[67] Tejerina H, De Paepe P, Closon MC, Van Dessel P, Darras C, Unger JP (2014). Forty years of USAID health cooperation in Bolivia. A lose–lose game?. Int J Health Plann Manag.

[68] Tejerina H, De Paepe P, Soors W, Lanza OV, Closon MC, Van Dessel P, Unger JP (2011). Revisiting health policy and the World Bank in Bolivia. Global Soc Policy.

[69] Bourdieu P (2012). Sur l'État. Cours au Collège de France (1989–1992).

